# Supplementary material for: Repellency Mechanism of Natural Guar Gum-Based Film Incorporated with Citral against Brown Planthopper, Nilaparvata lugens (Stål) (Hemiptera: Delphacidae)
Source: Int J Mol Sci. 2022 Jan 11;23(2):758. doi: 10.3390/ijms23020758 (PMC8776237; doi:10.3390/ijms23020758)
Supplement: Supplementary file 1 [file ijms-23-00758-s001.zip › ╘¡╩╝╩2╛▌/Dilution curve.pdf]

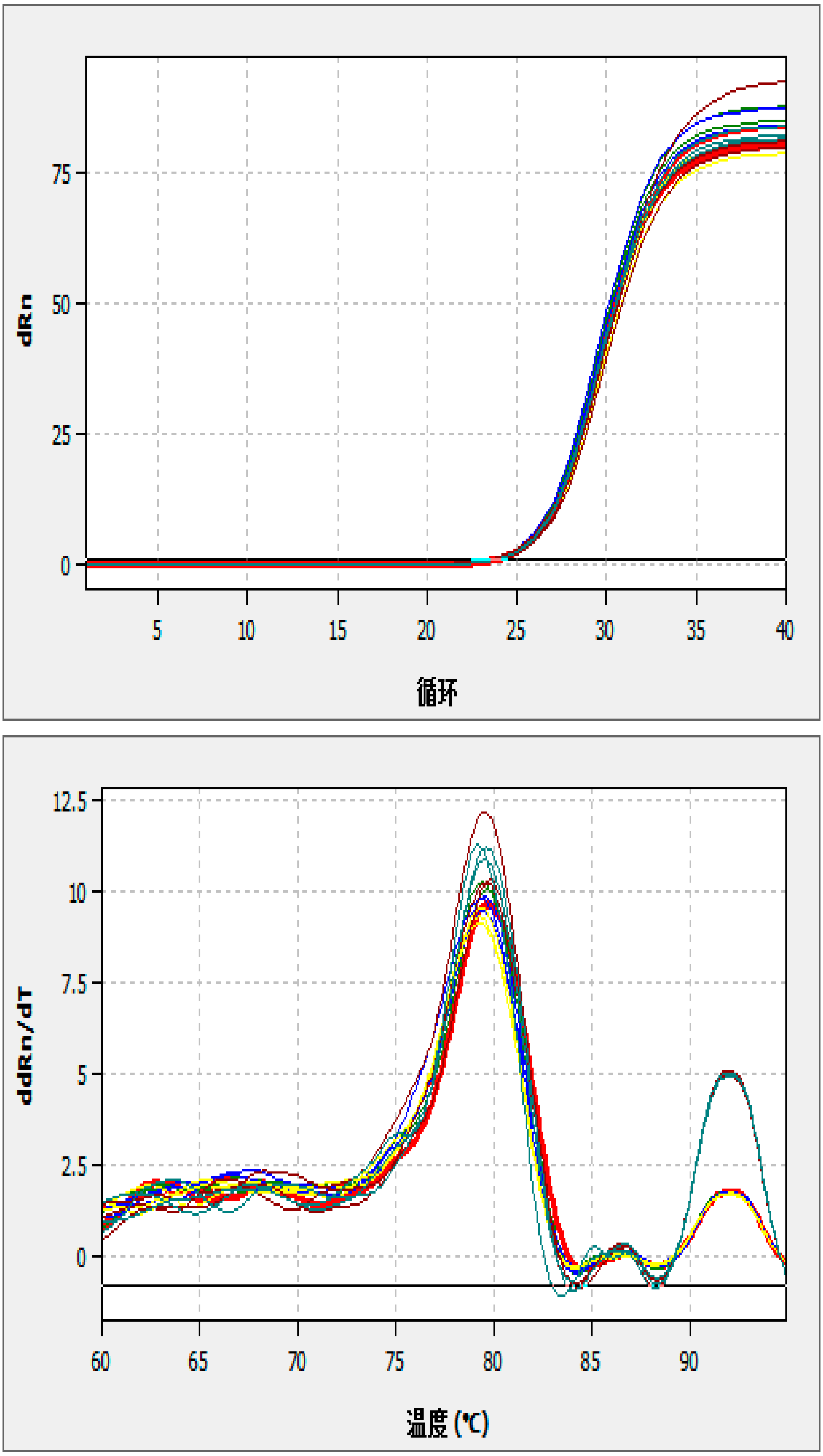

Fig 1 The amplification plot (up) and melting curve (down) of *U6 gene*

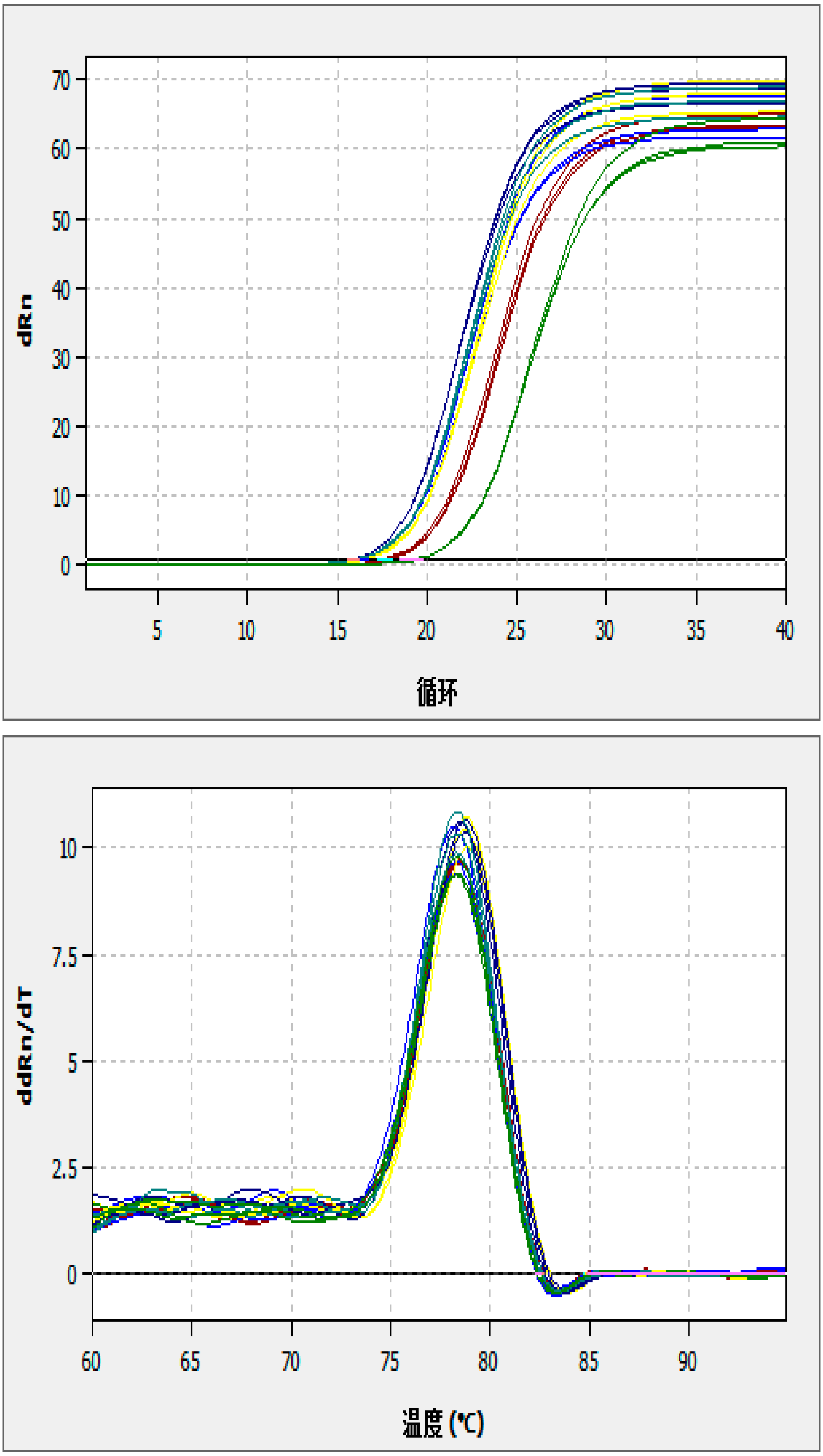

Fig 2 The amplification plot (up) and melting curve (down) of of *gene 797*

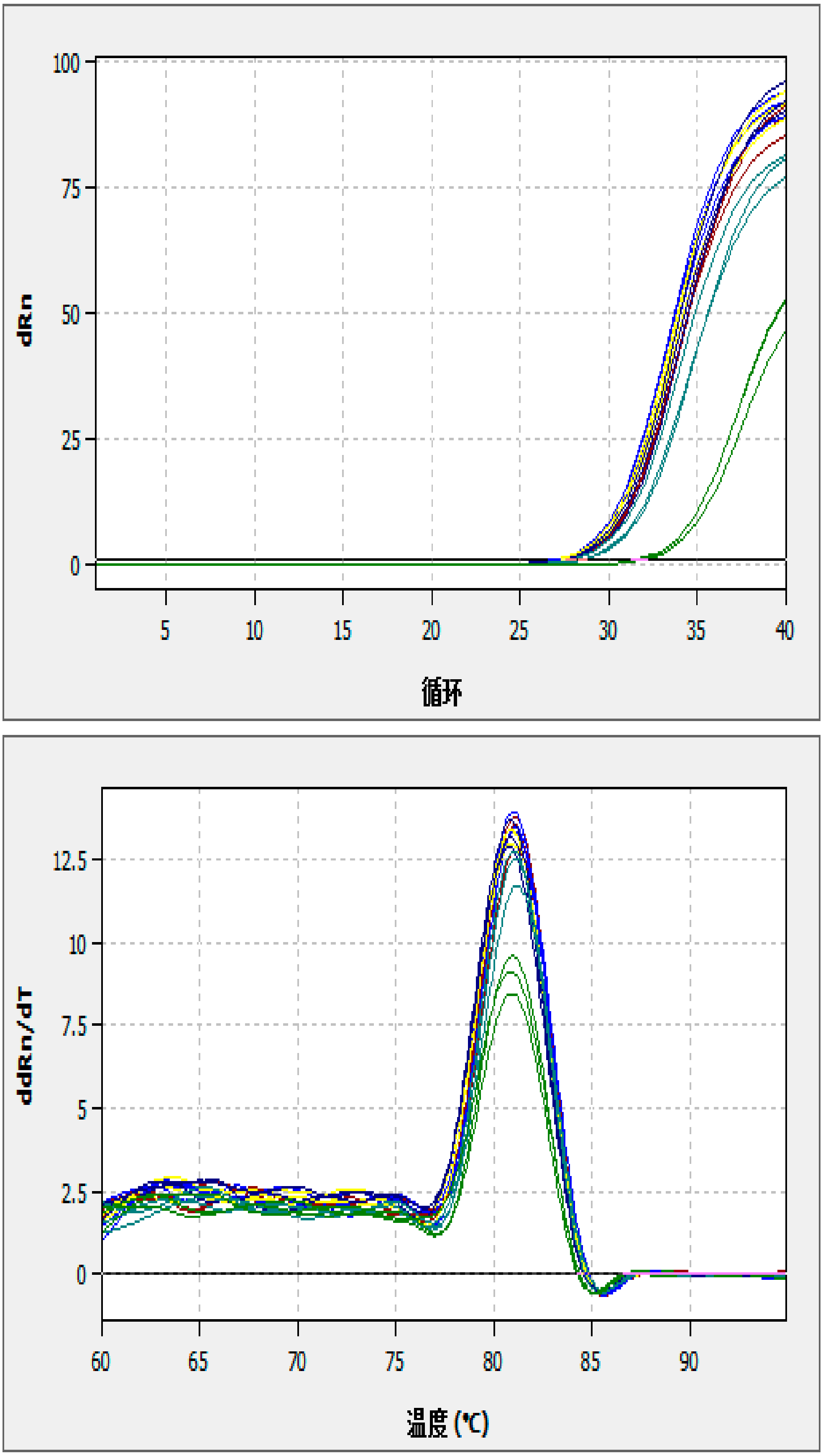

Fig 3 The amplification plot (up) and melting curve (down) of *gene 13110*
