# Supplementary figures and images for: Repellency Mechanism of Natural Guar Gum-Based Film Incorporated with Citral against Brown Planthopper, Nilaparvata lugens (Stål) (Hemiptera: Delphacidae)
Source: Int J Mol Sci. 2022 Jan 11;23(2):758. doi: 10.3390/ijms23020758 (PMC8776237; doi:10.3390/ijms23020758)

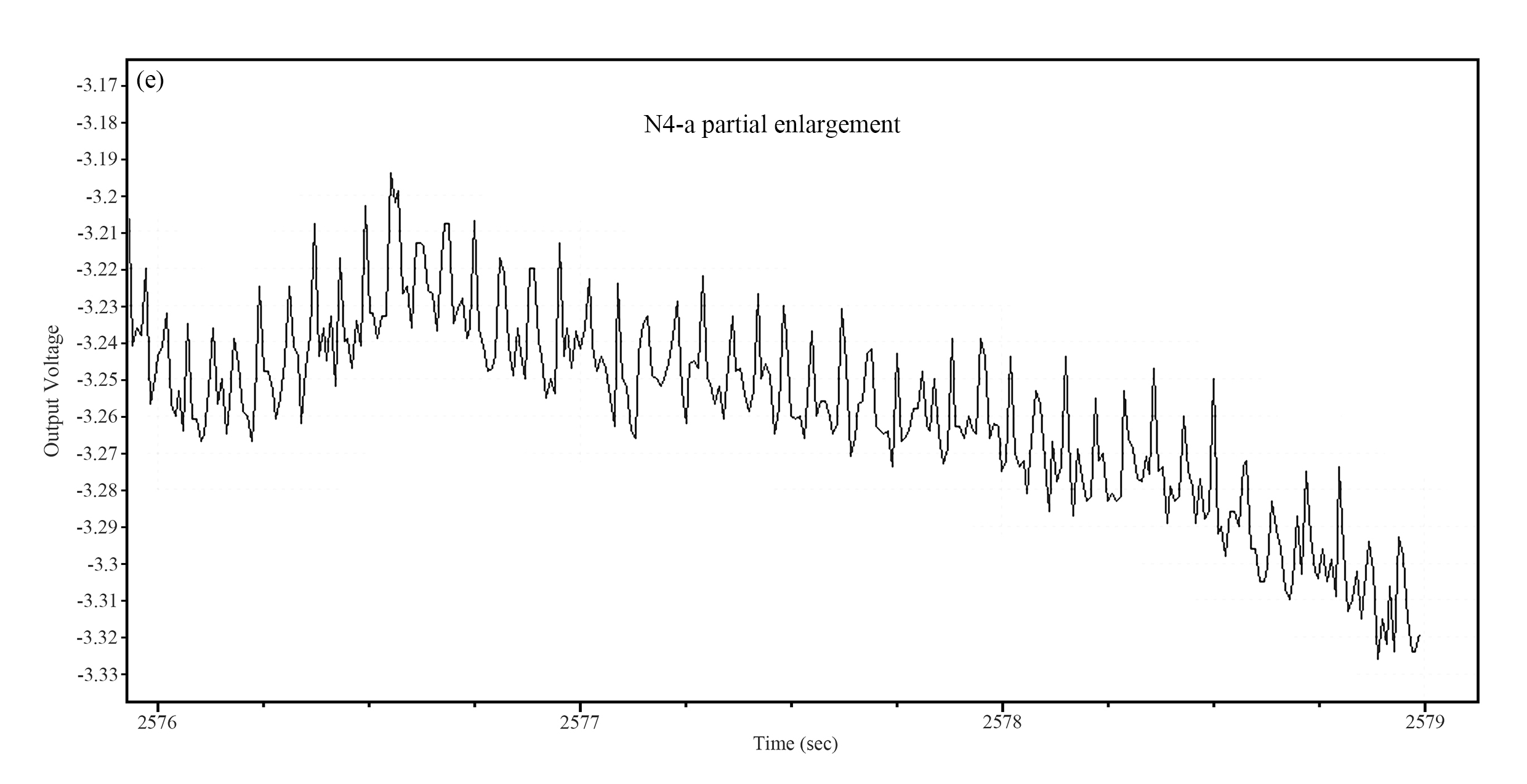

Supplement: Supplementary file 1 [file ijms-23-00758-s001.zip › ╘¡╩╝╩2╛▌/╬ó╨┼═╝╞1⁄4_20220107154434.jpg]

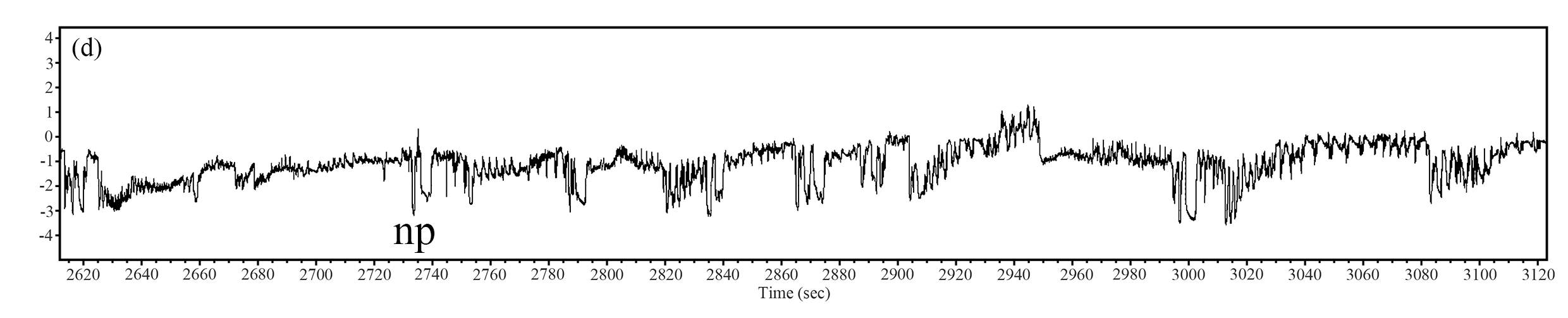

Supplement: Supplementary file 1 [file ijms-23-00758-s001.zip › ╘¡╩╝╩2╛▌/╬ó╨┼═╝╞1⁄4_20220107154448.jpg]

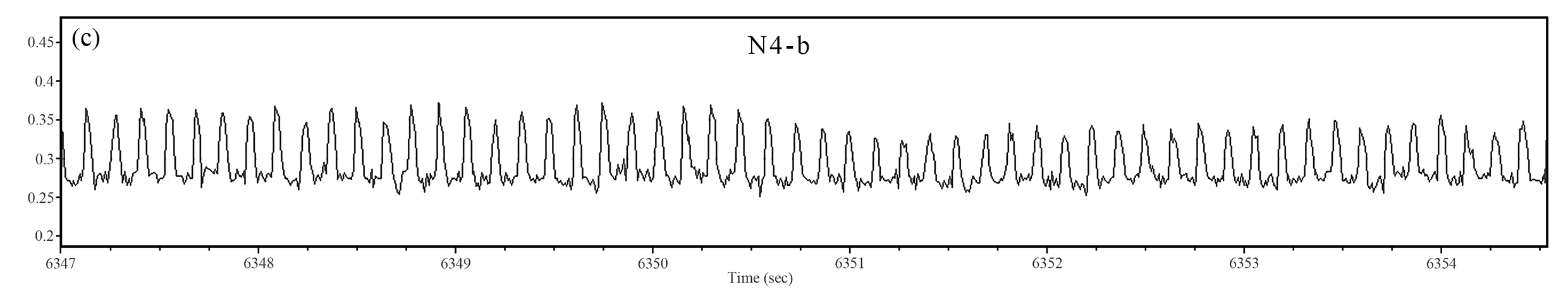

Supplement: Supplementary file 1 [file ijms-23-00758-s001.zip › ╘¡╩╝╩2╛▌/╬ó╨┼═╝╞1⁄4_20220107154459.jpg]

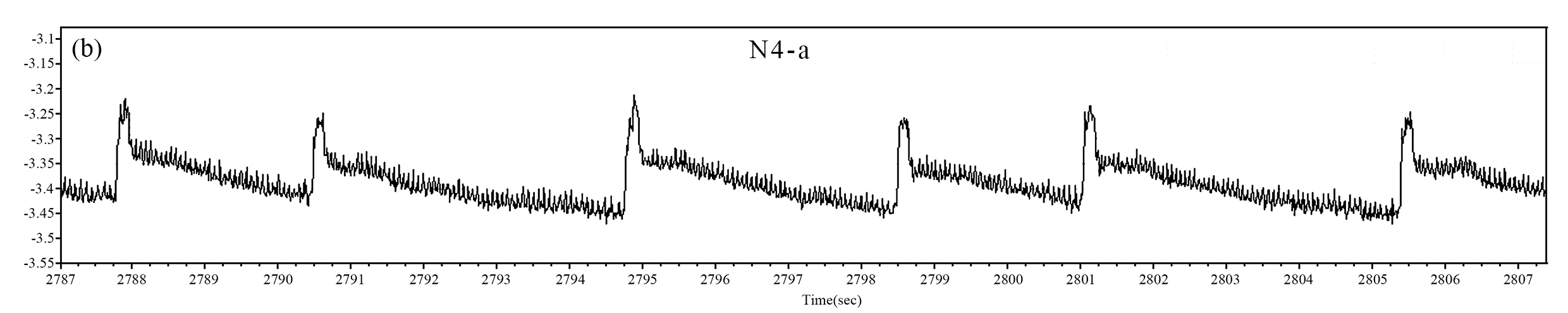

Supplement: Supplementary file 1 [file ijms-23-00758-s001.zip › ╘¡╩╝╩2╛▌/╬ó╨┼═╝╞1⁄4_20220107154508.jpg]

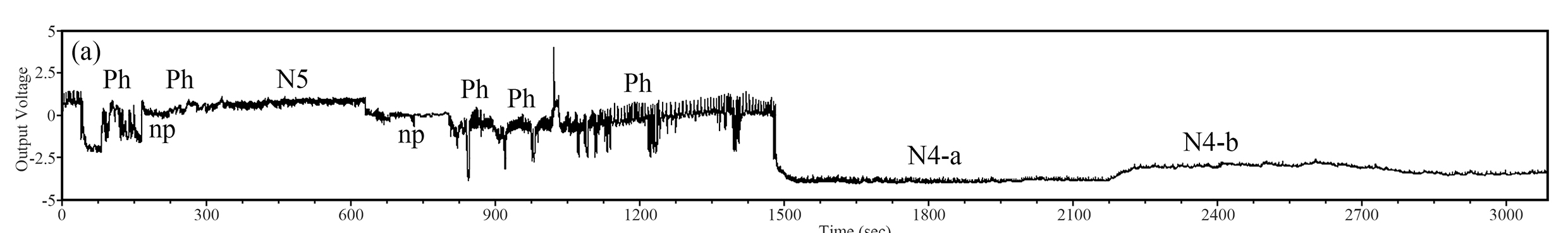

Supplement: Supplementary file 1 [file ijms-23-00758-s001.zip › ╘¡╩╝╩2╛▌/╬ó╨┼═╝╞1⁄4_20220107154519.jpg]

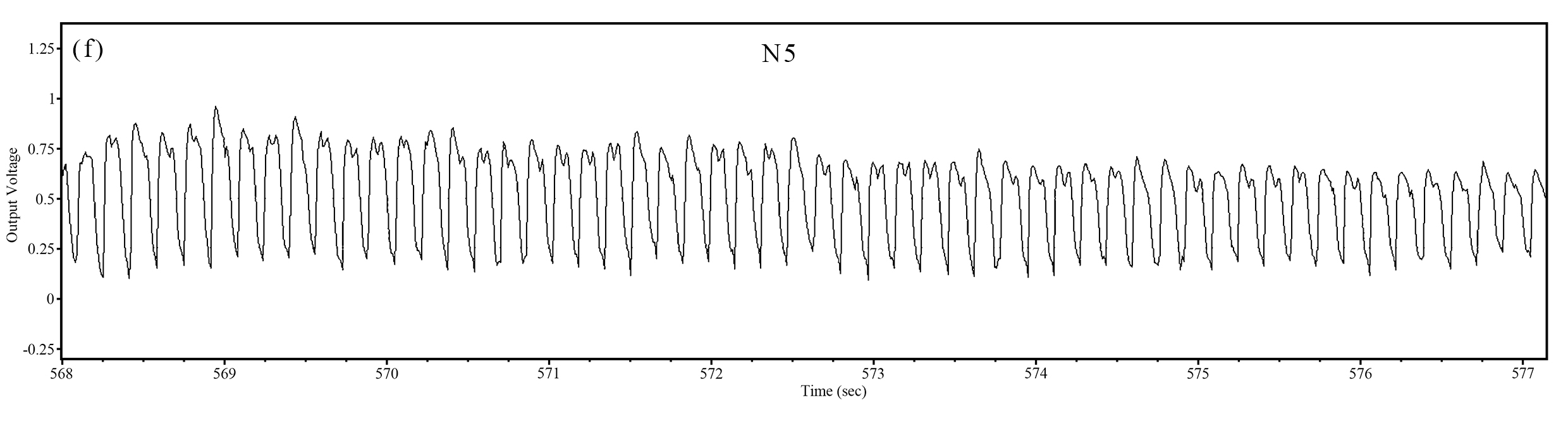

Supplement: Supplementary file 1 [file ijms-23-00758-s001.zip › ╘¡╩╝╩2╛▌/╬ó╨┼═╝╞1⁄4_20220107154527.jpg]
